# Perceptions of Patient Safety Culture and Medication Error Reporting among Early- and Mid-Career Female Nurses in South Korea

**DOI:** 10.3390/ijerph18094853

**Published:** 2021-05-01

**Authors:** Sun-Joo Jang, Haeyoung Lee, Youn-Jung Son

**Affiliations:** Red Cross College of Nursing, Chung-Ang University, Seoul 06974, Korea; icedcoffee@cau.ac.kr (S.-J.J.); im0202@cau.ac.kr (H.L.)

**Keywords:** nurses, patient safety culture, medication errors, expertise, nursing

## Abstract

Reporting medication errors is crucial for improving quality of care and patient safety in acute care settings. To date, little is known about how reporting varies between early and mid-career nurses. Thus, this study used a cross-sectional, secondary data analysis design to investigate the differences between early (under the age of 35) and mid-career (ages 35–54) female nurses by examining their perceptions of patient safety culture using the Korean Hospital Survey on Patient Safety Culture (HSPSC) and single-item self-report measure of medication error reporting. A total of 311 hospital nurses (260 early-career and 51 mid-career nurses) completed questionnaires on perceived patient safety culture and medication error reporting. Early-career nurses had lower levels of perception regarding patient safety culture (*p* = 0.034) compared to mid-career nurses. A multiple logistic regression analysis showed that relatively short clinical experience (<3 years) and a higher level of perceived patient safety culture increased the rate of appropriate medication error reporting among early-career nurses. However, there was no significant association between perception of patient safety culture and medication error reporting among mid-career nurses. Future studies should investigate the role of positive perception of patient safety culture on reporting errors considering multidimensional aspects, and include hospital contextual factors among early-, mid-, and late-career nurses.

## 1. Introduction

Patient safety is recognized as a pivotal component of healthcare quality, and is receiving global attention to prevent adverse events in healthcare settings [1,2]. Delivering safe care helps reduce adverse outcomes, such as longer hospital stays, high risk of mortality, and high medical costs in the care of patients [2,3,4]. In this regard, the significance of achieving a positive patient safety culture and patient safety has been continuously emphasized [5,6]. Patient safety culture is a crucial part of organizational culture in which healthcare providers recognize patient safety performance as the highest priority measure to prevent patient harm [3,7]. Furthermore, patient safety culture includes the manner in which healthcare providers are expected to behave, what attitudes and activities are appropriate, and what patient safety procedures are rewarded or prohibited [8,9,10]. Establishing robust patient safety culture has been found to significantly reduce the number of medical errors reported in healthcare organizations, as well as rates of re-admission and infection [11,12,13]. In contrast, poor patient safety can affect the quality of medical care, particularly error reporting [11,12,14].

Medication errors have significant implications for patient safety, which can lead to harm to patients and additional medical costs [14,15]. Medication errors are among the most common healthcare errors in Korea and worldwide [8,12,16,17]. Previous studies have reported that rates of medication errors vary from 3.3% to 53% owing to different definitions, settings, and methodologies in identification [3,14,15]. In South Korea, one study suggested that the electronic incident reporting systems of 50.8% of hospitals reported medication errors in acute care hospital settings [18]. Another study suggested that 63.6% of patients reported medication errors in acute care hospital settings [19]. Under-reporting or lack of emphasis on accurate medication error-reporting may be one of the major issues for the variation in the medication error rates [12,20]. Undoubtedly, low and inaccurate reporting of medication errors interferes with the identification and prevention of causes, limiting opportunities to improve patient safety by understanding and sharing knowledge on error detection and safe practices [11,16]. Hence, reporting is extremely important in preventing medication errors, a crucial step for encouraging safe practices, including medication safety [14].

To date, at the organizational level, hospitals strive to improve quality of care through practices such as ensuring a positive patient safety climate, reducing workload and time pressure, providing education, and following patient safety protocols [3,16]. However, hesitation in reporting errors at the individual level remains a significant barrier to ensuring patient safety [21,22]. Fear of being blamed was considered the main barrier to reporting medication errors [6]. Prior studies have reported that approximately 50–96% of adverse events are not reported because of the fear of creating a negative impression and being punished [11,23]. Additionally, a lack of adequate support from colleagues or supervisors may be another barrier to error reporting [12,24]. As registered nurses are front-line healthcare providers, who spend more time in direct patient care than other medical staff in the hospital, they play a vital role in ensuring high-quality and safe care [11,25]. In particular, nurses are mainly responsible for administering medication and, on average, they spend about 40% of their time on medication management [15]. Therefore, nurses have a higher chance of making medication errors because of the nature of their work [14,26].

The high turnover rate of nurses and low nurse staffing levels are a serious threat to patient safety [27]. The shortage of nursing staff in hospitals is a global issue, and South Korea is no exception [28,29]. According to a report by the Korean Hospital Nurses Association (2018) [30], Korean nurses had a turnover rate of 15.4%, in general, and 45.5% among nurses with less than one year of experience. Thus, clinically inexperienced nurses, who are still learning their roles, are more prone to making medication errors than experienced nurses [31,32]. Some evidence has revealed that early-career nurses seek employment in nursing environments with a focus on quality patient care [33,34,35]. Despite the importance of error communication, the actual amount of error reporting is lower than the number of actual medication errors determined by experts [11,20]. Thus, understanding perceived barriers to error reporting, from the nurses’ perspective, is crucial in promoting reporting behavior.

As nurses are fundamental for improving patient safety, they should be able to freely address the adverse events that might occur due to human error, as well as the problems in the healthcare system [2,3]. Since the Patient Safety Act in Korea was announced in July 2016, healthcare institutions have appointed employees to oversee patient safety and to facilitate the reporting of patient safety incidents [36]. Although the error reporting system is the most commonly used method for identifying medication errors, significant under-reporting still persists in Korea and across the world [19,37]. Moreover, few studies have examined the relationship between nurses’ perceptions of patient safety culture and medication error reporting in Korea. We hypothesized that there would be a difference between early-career (aged under 35 years) and mid-career nurses (aged 35–54 years), as defined by the World Health Organization [38], regarding the perceived patient safety culture and the rate of medication error reporting. Thus, this study compares the association between perception of patient safety culture and medication error reporting among early- and mid-career female nurses. In addition, it addresses the impact of patient safety culture on medication error reporting among early-career nurses who are inexperienced compared to mid-career nurses.

## 2. Materials and Methods

### 2.1. Study Design and Participants

This is a secondary analysis of existing data of 311 nurses of a tertiary hospital collected from March to May 2015, to examine patient safety culture and adverse nursing outcomes [39]. From the existing dataset, nurses under 35 were classified as early-career nurses and those aged 35 or older were classified as mid-career nurses [38]. Data from 260 early-career and 51 mid-career nurses were included. The sample size for multiple regression was determined using the G*power 3.1.9.2 version software [40], and the minimum sample size was calculated to be 172 with a significance of 0.05, power of 0.95, 10 predicting variables, and effect size (f^2^) of 0.15 (medium). Thus, a sample size of 260 was deemed sufficient.

### 2.2. Measures

Information about participants’ general characteristics (age, education level, marital status) and work-related characteristics (total clinical career, current place of work, average weekly work hours, participation in patient safety culture education, participation in campaigns) was collected using a questionnaire. The instruments used to assess perceived patient safety culture, the main independent variable, and medication error reporting, the dependent variable, are described below.

#### 2.2.1. Perceived Patient Safety Culture

The Korean [41] Hospital Survey on Patient Safety Culture (HSPSC), originally developed by the Agency for Healthcare Research and Quality (2004) [42], was used to assess nurses’ perceived patient safety culture. This is a 43-item instrument, comprising six domains, and rated on a 5-point Likert scale (1: not at all-5: always, total score: 43–215). A higher score indicates greater perceived patient safety culture. The Cronbach’s α of the Korean version of the HSPSC was 0.67–0.84 at the time of adaptation and 0.81 in this study.

#### 2.2.2. Medication Errors Reporting

In the medication error reporting data previously collected using a 5-point Likert scale (1 = never, 2 = rarely, 3 = occasionally, 4 = most of the time, 5 = always), a score of 5 was considered appropriate medication error reporting, and a score of less than 5 was considered to be inappropriate.

### 2.3. Data Analysis

Data were analyzed using IBM SPSS Statistics software (version 26.0). General and work-related characteristics, perceived patient safety culture, and medication error reporting of participants were analyzed using descriptive statistics. The differences in perceived patient safety culture and medication error reporting, according to general and work-related characteristics, were analyzed using an independent *t*-test, a one-way ANOVA, and the Chi-squared test. Predictors of medication error reporting were identified using logistic regression analysis. The goodness of fit of the regression model was verified using the Hosmer–Lemeshow test.

### 2.4. Ethical Considerations

This study was approved for secondary data analysis by the institutional review board of Chung-Ang University (1041078-202103-HRSB-056-01).

## 3. Results

### 3.1. Participants’ General and Work-Related Characteristics

All the participants were female, and 260 (83.6%) were early-career nurses. The mean age of early-career nurses was 27.40 years (SD 3.39), and 78.1% of them were single. The mean total clinical career length was 63.92 months (SD 40.06), and weekly average work hours, 45.65. The mean age of the mid-career nurses was 40.10 years (SD 2.37), and 84.3% of them were married. The mean total clinical career was 210.96 months (SD 33.67), and weekly average work hours was 43.86 (Table 1).

As shown in Table 2, among early-career nurses, 95.0% and 82.7% had participated in a safety education and safety culture campaign, respectively, and 29.6% engaged in appropriate medication error reporting. In mid-career nurses, 94.1% and 98.0% had participated in a safety education and safety culture campaign respectively, and 37.3% engaged in appropriate medication error reporting. While there were no significant differences in the rate of participation in safety education between the two groups, the rate of participation in a safety culture campaign was higher among mid-career nurses (t = 7.97, *p* = 0.004). The mean perceived patient safety culture score in early-career nurses was 146.97, lower than that of mid-career nurses (t = 2.13, *p* = 0.034). However, there were no significant differences in medication error reporting between the two groups.

### 3.2. Differences in Medication Error Reporting According to the Characteristics of Early-Career Nurses

Among early-career nurses, medication error reporting did not differ significantly by general characteristics, work hours, work unit, participation in safety education, or participation in safety culture campaigns. However, a significant statistical difference was found regarding total clinical career (χ^2^ = 7.45, *p* = 0.024) and perceived patient safety culture (χ^2^ = 10.61, *p* = 0.002) (Table 3).

### 3.3. Logistic Regression Analysis for Variables Predicting Appropriate Medication Error Reporting

For variable selection, the enter method is a procedure in which all variables in a block are entered in a single step [43]. To identify the predictors of medication error reporting in early-career nurses, logistic regression was performed using the enter method, and the regression model was significant (χ^2^ = 20.12, *p* = 0.017). The variation inflation factor (VIF) was below 10.00, with a range of 1.04–1.27, verifying the absence of multicollinearity among the independent variables. Total clinical career and patient safety culture were identified as significant predictors of medication error reporting. Compared to those with a total clinical career of less than three years, the likelihood of engaging in appropriate medication error reporting was 0.39 for those with a clinical career of 3–4 years and 0.49 for those with a clinical career of five years or longer. This was 2.04 times more likely than those with a career of three years, 2.56 times more likely than those with a career of 3–4 years, and 2.04 times more likely than those with a career of five years or longer to engage in appropriate medication error reporting. The likelihood of engaging in appropriate medication error reporting increased 2.44 times among those with a high patient safety culture score (higher than the median 147) (Table 4). Logistic regression analysis was performed to identify the predictors of medication error reporting in mid-career nurses. While the VIF was below 10.00, with a range of 1.06–1.68, confirming the absence of multicollinearity among the independent variables, the regression model was not significant (χ^2^ = 12.53, *p* = 0.129).

## 4. Discussion

This study compared the differences in perceived patient safety culture and medication error reporting between early- and mid-career nurses, to identify the predictors of appropriate medication error reporting. In particular, there was a focus on identifying the predictors of medication error reporting in early-career nurses, who are at a higher risk of medication errors and have a higher turnover.

The logistic regression identified total clinical career and patient safety culture scores as significant predictors of appropriate medication error reporting in early-career nurses. Nurses with a career of three years were 2.56 times more likely to report errors than nurses with a career of 3–4 years, and 2.04 times more likely than nurses with a career of five years or longer. The likelihood of engaging in appropriate medication error reporting increased 2.44 times among those with a high patient safety culture score (equal to or higher than the median 147). In other words, appropriate medication error reporting is more common among early-career nurses with a clinical career of less than three years and a higher perceived patient safety culture score. The findings of our study are similar to the findings of a previous study [44] where nurses with a higher perceived patient safety culture were more engaged in appropriate medication error reporting. However, our results showing that early-career nurses with a total clinical career of less than three years were more likely to report medication errors, in contrast to previous results [44]. Direct comparison is difficult because different types of medication errors are not taken into account, and the rate of medication error reporting varies substantially according to the definition, setting, and methods of assessing medication error [3,14,15]. Moreover, the reporting of patient safety incidents differs according to the severity of the harm caused by the event (near miss, adverse event, sentinel event) [44]. It is also not possible to completely exclude the impact of biases during the measurement of medication errors. Furthermore, the fact that nurses with a shorter clinical career are more likely to experience medication errors than those with longer clinical experiences [31,32] may be explained by the fact that early-career nurses are more frequently involved in medication errors and thus report more patient safety incidents. To lower medication errors, the medication administering competency of early-career nurses should be fostered through training. The high rate of medication error reporting, even when nurses may choose not to report errors, is promising as it indicates that early-career nurses are aware of the importance of error reporting. Thus, education and support to continuously foster error reporting may be necessary.

There were no significant differences in appropriate medication error reporting by early-career nurses according to their general characteristics, work hours, work unit, participation in safety education, and participation in safety culture campaigns. However, appropriate medication error reporting significantly differed according to nurses’ total clinical career and perceived patient safety culture. The appropriate medication error reporting group consisted of a relatively higher percentage of nurses with a clinical career of less than three years, while the inappropriate medication error reporting group consisted of a higher percentage of nurses with a clinical career of five years or more. In addition, the appropriate medication error reporting group consisted of a higher percentage of nurses with a higher perceived patient safety culture score. Since this study employed a cross-sectional design, it is difficult to draw conclusions on the causality of the relationship; that is, whether nurses with high perceived patient safety culture actually report medication errors more appropriately. Furthermore, the number of medication errors reported in this study was assessed by self-reporting, and not the actual number of medication errors. Therefore, it would be important to have longitudinal studies that have access to the actual number of medication errors, reporting rates, and follow-ups, in order to investigate the causal relationship between appropriate medication error reporting and level of perceived patient safety culture. Although the rate of participation in safety education did not differ between early- and mid-career nurses, more mid-career nurses participated in a safety culture campaign compared to early-career nurses. Mid-career nurses also had a higher perceived patient safety culture score, but medication error reporting did not significantly differ between the two groups. Participation rates in patient safety education and patient safety culture campaigns were high in both groups.

South Korea launched the healthcare institution accreditation system in 2010 with an amendment to the Medical Service Act, and enacted the Patient Safety Act in 2016, establishing a patient safety reporting system and laying the foundation for systematic governmental management of patient safety issues [45]. This is speculated to be the reason for the high participation in patient safety education and safety culture campaigns among nurses. However, the fact that only 29.6% of early-career nurses and 37.3% of mid-career nurses engaged in appropriate medication error reporting despite the overall high rate of participation suggests that there are barriers to reporting patient safety incidents, such as medication errors, regardless of knowledge or perception of patient safety. The purpose of this study was not to identify the barriers to reporting experienced by nurses. However, it is hypothesized that factors such as fear of being punished [6], lack of adequate support from colleagues or supervisors [12,24], and inadequate managerial feedback [11] may have been some of the major barriers. Furthermore, as previous studies identified, difficulties in filling in forms, and lack of knowledge regarding medical error reporting systems could also act as barriers [26]. It is important to assess if nurses are well-informed about the system for reporting medication errors and if there are any problems in using the reporting system to address these issues. Reporting medication errors is as important as preventing them and is a critical process in establishing safe practice [14]. Systematizing active error reporting and learning from the errors may be the most effective strategy to reduce patient safety incidents [45]. In addition to increasing awareness and knowledge about patient safety, through patient safety education and campaigns, organizations should strive to cultivate a culture that promotes the use of an error reporting system and active discussion of points for improvement based on these errors. Patient safety culture reduces stress associated with secondary damage, increases organizational support, and contributes to lowering turnover and absenteeism [46]. Organizations should strive to establish and promote a desirable patient safety culture. Additionally, reporting culture influences the rate of incident reporting, nursing safety practice and perception of work. Thus, to encourage voluntary error reporting, lead nurses should ensure that workload is at an appropriate level, and devise strategies to boost job satisfaction [37].

Although a high turnover rate for nurses is a factor that threatens patient safety [27], a serious shortage of nursing staff in hospitals remains a major problem in Korea [29]. The fear of being blamed for a mistake is a major barrier to reporting patient safety incidents, including medication errors [6]. Another reported barrier is the lack of appropriate support from colleagues and managers [12,24]. Post-traumatic stress disorder (PTSD) is a psychological trauma caused by exposure to a traumatic event such as patient safety incidents [47,48]. Sometimes, nurses experience PTSD symptoms such as fear, loss of self-esteem, guilt, anger, burnout, and frustration after being involved in medication errors [47,48,49]. Without proper treatment, these symptoms result in fatigue, depression, and reduced empathy, increasing the risk of more errors [50,51], and potentially leading to turnover and absenteeism, ultimately affecting the organization [46,52,53]. Hence, by analyzing the causes and identifying ways to prevent future errors in patient safety incidents (e.g., medication errors), increasing support from colleagues, managers, and the institution, and conducting quality improvement activities, we can help overcome trauma in nurses who have experienced patient safety incidents. A report on global nursing policy [38] emphasizes the need to reduce burnout and turnover among early-career nurses. Early-career nurses experience burnout symptoms at least once in the first three years of their working life [35], and early turnover leads to a serious shortage of nursing staff [28,29], which, in turn, threatens patient safety [27]. To protect nurses and patients from medication errors, it is important to implement practical measures by meticulously analyzing and eliminating the barriers to medication error reporting and conducting well-designed studies for this purpose.

This study had several limitations. First, all participants were female, and 83.6% were early-career nurses. Moreover, the study data were collected from a single hospital, resulting in possible selection bias. Subsequent studies should include male nurses and more mid or late-career nurses in order to reduce sampling bias. Second, we only included self-reported, individual-level parameters in the analysis and could not investigate organizational and ward-related characteristics. Subsequent studies should examine multidimensional aspects, including hospital contextual factors, among early-, mid-, and late-career nurses. Third, medication error reporting was assessed via self-reports and not based on the actual data reported in the system. Thus, future studies should contrast this data with objective data on medication error reporting to identify its predictors. Fourth, this study was cross-sectional with a small sample size, which can limit generalizability. Furthermore, it is difficult to establish the causal relationship between medication error reporting and its predictors. In the future, a longitudinal study that addresses these limitations should be designed to more accurately identify the above-mentioned factors. Despite these limitations, this study is significant as the first attempt to analyze nurses’ perceived patient safety culture and medication error reporting, by classifying them into early-career and mid-career nurses, based on their length of career.

## 5. Conclusions

We found that early-career nurses with a high patient safety culture are nearly 2.4 times more likely to report medication error compared to those with a low patient safety culture. For mid-career nurses, patient safety culture was not associated with medication error reporting. Regardless of nurses’ career, the rate of appropriate medication error reporting was still low (29.6% of ear-career nurses and 37.3% of mid-career nurses). It highlights that there are still barriers that hinder medication error reporting. To prevent patient safety incidents, including medication errors, under-reporting of patient safety incidents must be addressed. This would require systematic and institutional amelioration to facilitate proper reporting of patient safety incidents. Additionally, a patient safety culture that promotes active discussions on the causes of problems and potential measures for improvement needs to be established. Early-career nurses lack experience and are at a relatively higher risk of being involved in patient safety incidents. On the other hand, more experienced nurses including mid-career nurses are usually given more serious patient cares, which can encounter more patient safety incidents than their younger counterparts. Therefore, nurse managers or administrators should consider work stressors and work performance according to nurses’ career.

## Figures and Tables

**Table 1:** General and work-related characteristics (*n* = 311).

Characteristics	Category	Early-Career Nurses (*n* = 260)	Mid-Career Nurses (*n* = 51)	t/F or χ^2^	*p*-Value
		*n* (%)/M (SD)	*n* (%)/M (SD)		
Age (years)		27.40(3.39)	40.10(2.37)	25.56	<0.001
Marital status	Single	203(78.1)	8(15.7)	76.08	<0.001
	Married	57(21.9)	43(84.3)		
Education level	Bachelor’s	250(96.2)	34(66.7)	54.02	<0.001
	Master’s	10(3.8)	17(33.3)		
Total clinical career		63.92(40.06)	210.96(33.67)	24.56	<0.001
Average weekly work hours	<50	127(48.8)	36(70.6)	8.08	0.005
	≥50	133(51.2)	15(24.3)		
Current work unit	Medical ward	80(30.8)	10(19.6)	7.96	0.051
	Surgical ward	74(28.5)	17(33.3)		
	ICU	68(26.2)	14(27.5)		
	etc.	38(14.6)	10(19.6)		

M: mean, SD: standard deviation, ICU: intensive care unit. etc.: emergency department, pediatrics, obstetrics and gynecology unit.

**Table 2:** Comparison of patient safety culture-related variables between early- and mid-career nurses.

Characteristics	Category	Early-Career Nurses (*n* = 260)	Mid-Career Nurses (*n* = 51)	t/F or χ^2^	*p*-Value
		*n* (%)/M (SD)	*n* (%)/M (SD)		
Safety education	Yes	247(95.0)	48(94.1)	0.07	>0.999
	No	13(5.0)	3(5.9)		
Safety culture campaign	Yes	215(82.7)	50(98.0)	7.97	0.004
	No	45(17.3)	1(2.8)		
Perceived patient safety culture		146.97(14.30)	151.59(13.29)	2.13	0.034
Medication error reporting	Appropriate	77(29.6)	19(37.3)	1.17	0.320
	Inappropriate	183(70.4)	32(62.7)		

M: mean, SD: standard deviation.

**Table 3:** Differences in medication error reporting according to the characteristics of early-career nurses (*n* = 260).

Characteristics	Category	*n* (%)		χ2	*p*-Value
		Appropriate MER (*n* = 77)	Inappropriate MER (*n* = 183)		
Marital status	Single	61(79.2)	142(77.6)	0.08	0.870
	Married	16(20.8)	41(22.4)		
Education level	Bachelor’s	76(98.7)	174(95.1)	1.92	0.290
	Master’s	1(1.3)	9(4.9)		
Total clinical career (years)	<3	35(45.4)	52(28.4)	7.45	0.024
	3–4	10(13.0)	38(20.8)		
	≥ 5	32(41.6)	125(50.8)		
Average weekly work hours	<50	38(49.4)	89(48.6)	0.01	>0.999
	≥50	39(50.6)	94(51.4)		
Current work unit	Medical ward	23(29.9)	57(31.1)	3.62	0.305
	Surgical ward	21(27.3)	53(29.0)		
	ICU	17(22.1)	51(27.9)		
	etc.	16(20.8)	22(12.0)		
Safety education	Yes	73(94.8)	174(95.1)	0.01	>0.999
	No	4(5.2)	9(4.9)		
Safety culture campaign	Yes	63(81.8)	152(83.1)	0.06	0.858
	No	14(18.2)	31(16.9)		
Perceived patient safety culture	Low	28(36.4)	107(58.5)	10.61	0.002
	High	49(63.6)	76(41.4)		

ICU: intensive care unit, MER: medication error reporting, etc.: emergency department, pediatrics, obstetrics and gynecology unit.

**Table 4:** Factors influencing appropriate medication error reporting (*n* = 311).

Early-Career Nurses (*n* = 260)			Mid-Career Nurses (*n* = 51)		
Variables	AOR (95% CI)	*p-*Value	Variables	AOR (95% CI)	*p*-Value
Marital status			Marital status		
Single	1		Single	1	
Married	1.24(0.58–2.66)	0.576	Married	1.94(0.31–11.97)	0.477
Education level			Education level		
Bachelor’s	1		Bachelor’s	1	
Master’s	0.28(0.03–2.43)	0.249	Master’s	3.45(0.78–15.26)	0.136
Total clinical career (years)			Total clinical career (years)		
1–2	1		8–14	1	
3–4	0.39(0.17–0.93)	0.033	15–22	11.08(0.67–183.87)	0.093
5–15	0.49(0.25–0.95)	0.034			
Clinical work unit			Clinical work unit		
Ward	1		Ward	1	
Other	1.23(0.68–2.22)	0.488	Other	2.18(0.55–8.69)	0.27
Average weekly work hours			Average weekly work hours		
<50	1		<50	1	
≥50	1.19(0.67–2.12)	0.543	≥50	5.57(0.99–31.31)	0.051
Patient safety education			Patient safety education		
Yes	1		Yes	1	
No	0.81(0.22–2.95)	0.75	No	10.54(0.27–417.69)	0.21
Campaign			Campaign		
Yes	1		Yes	1	
No	1.38(0.64–2.97)	0.416	No	0.00(0.00–0.00)	>0.999
Perceived patient safety culture			Perceived patient safety culture		.
Low (below 147)	1		Low (below 147)	1	
High (≥147)	2.44(1.38–4.33)	0.002	High (≥147)	1.74(0.42–7.23)	0.446
χ^2^ = 20.12, *p* = 0.017, −2 Log Likelihood χ^2^ = 295.82,			χ^2^ = 12.53, *p* = 0.129, −2 Log Likelihood χ^2^ = 54.82,		
Cox and Snell R^2^ = 0.07, Nagelkerke R^2^ = 0.11			Cox and Snell R^2^ = 0.22, Nagelkerke R^2^ = 0.30		
Hosmer and Lemeshow χ^2^ = 5.86, *p* = 0.663			Hosmer and Lemeshow χ^2^ = 7.66, *p* = 0.467		

AOR: adjusted odd ratio, CI: confidence interval.

## Data Availability

The data presented in this study are available on request from the corresponding author and with permission of the Institutional Review Board of Chung-Ang University.
